# peptidy: a light-weight Python library for peptide representation in machine learning

**DOI:** 10.1093/bioadv/vbaf058

**Published:** 2025-03-21

**Authors:** Rıza Özçelik, Laura van Weesep, Sarah de Ruiter, Francesca Grisoni

**Affiliations:** Department of Biomedical Engineering, Institute for Complex Molecular Systems, Eindhoven University of Technology, Eindhoven 5612AZ, Netherlands; Centre for Living Technologies, Alliance TU/e, WUR, UU, UMC Utrech, Utrecht 3584CB, Netherlands; Department of Biomedical Engineering, Institute for Complex Molecular Systems, Eindhoven University of Technology, Eindhoven 5612AZ, Netherlands; Department of Biomedical Engineering, Institute for Complex Molecular Systems, Eindhoven University of Technology, Eindhoven 5612AZ, Netherlands; Department of Biomedical Engineering, Institute for Complex Molecular Systems, Eindhoven University of Technology, Eindhoven 5612AZ, Netherlands; Centre for Living Technologies, Alliance TU/e, WUR, UU, UMC Utrech, Utrecht 3584CB, Netherlands

## Abstract

**Motivation:**

Peptides are widely used in applications ranging from drug discovery to food technologies. Machine learning has become increasingly prominent in accelerating the search for new peptides, and user-friendly computational tools can further enhance these efforts.

**Results:**

In this work, we introduce peptidy—a lightweight Python library that facilitates converting peptides (expressed as amino acid sequences) to numerical representations suited to machine learning. peptidy is free from external dependencies, integrates seamlessly into modern Python environments, and supports a range of encoding strategies suitable for both predictive and generative machine learning approaches. Additionally, peptidy supports peptides with post-translational modifications, such as phosphorylation, acetylation, and methylation, thereby extending the functionality of existing Python packages for peptides and proteins.

**Availability and implementation:**

peptidy is freely available with a permissive license on GitHub at the following URL: https://github.com/molML/peptidy.

## 1 Introduction

Peptides are relevant molecular entities in chemistry and biology, with applications ranging from drug discovery (Mangoni 2011, Sharma *et al.* 2023) to food technology (Hartmann and Meisel 2007, De Leon Rodriguez and Hemar 2020). Machine learning has accelerated peptide discovery, e.g. for *de novo* design, sequence optimization, and property/bioactivity prediction (Fjell *et al.* 2009, Lee *et al.* 2016, Grisoni *et al.* 2018, Lee *et al.* 2018).

A key step for machine learning is peptide representation (Jenssen 2011, Yue *et al.* 2023), whereby relevant structural information is converted into numerical formats for model training. Several strategies can be adopted to encode peptide information, e.g. via description of physicochemical features (Jenssen 2011), one-hot encoding (Erjavac *et al.* 2022), and/or evolutionary information (Eddy 2004). Each of these approaches captures different structural information, might be suited for different machine learning approaches (ElAbd *et al.* 2020), and might uniquely contribute to model performance (Grisoni *et al.* 2019, Erjavac *et al.* 2022). While public implementations are available for specific encoding methods (e.g. Osorio *et al.* 2015, Müller *et al.* 2017), bringing them together into a single project often requires dependency alignment and possible incompatibility hurdles.

Here, we introduce peptidy—a light-weight Python library that implements various peptide representations for machine learning. Key features of peptidy are the following:

- it has no external dependency and integrates smoothly into all modern Python environments;
- it encompasses a range of strategies for peptide encoding, useful for predictive and generative machine learning applications;
- it supports several post-translational modifications to amino acids, e.g. phosphorylation, acetylations, and methylation, extending the capabilities of existing Python packages.

Thanks to its light-weight character, peptidy is expected to accelerate the analysis of different peptide representation strategies for machine learning, and to be easy to adopt and expand upon. Comprehensive online documentation and user guides were developed to facilitate the adoption of peptidy by the scientific community. We expect peptidy to further contribute to the application of machine learning for peptide discovery and optimization.

## 2 peptidy

peptidy is a light-weight and easy-to-use Python package (v3.6 or greater) to convert amino acid sequences into ‘machine-learning-ready’ representations. Five popular peptide encoding methods are available in peptidy v0.0.1, with support for post-translational modifications in the sequences. The initial release of peptidy supports 20 standard amino acids and 8 post-translational modifications, extending the capabilities of existing peptide processing tools. All the available representations (except for global descriptors) are also suited for generative deep learning. This can be achieved by adding special elements to indicate the beginning and end of a given peptide sequence (see the technical documentation accompanying peptidy for more information). The implemented peptide representations are briefly described below and summarized in Table 1.

**Table 1.** Description of encoding methods in peptidy.^c^

Method	Information captured	Dimension
Peptide descriptors^a^	Captures 48 physicochemical properties of peptides as numeric values (descriptors). A full description of the available properties can be found in the technical documentation. The selection of a subset is possible.	48×1
Amino acid descriptors^a^^,^^b^	Encodes 18 physicochemical properties at the amino acid level (selection of a subset possible). A full description of the available properties can be found in the technical documentation.	L×18
BLOSUM62 encoding^a^^,^^b^	Represents amino acids in terms of their evolutionary similarity to each other and represents the peptide as the sequence of such vectors.	L×21
One-hot encoding^a^^,^^b^	Creates fixed vectors per amino acid type (where 1 indicates its presence in a specific position in the sequence, and 0 indicates its absence).	L×28
Label encoding^a^^,^^b^	Maps each amino acid to an integer (label) and represents the peptide as a sequence of labels.	L×1

a Supports post-translational modifications.

b Supports generative deep learning.

c For each approach, the chemical information captured and the output dimension are reported (L: number of amino acids in the sequence).

### 2.1 Peptide descriptors

peptidy implements a total of 48 global descriptors that capture physico-chemical properties (e.g. charge density, isoelectric point). Selecting a subset of descriptors is possible.

### 2.2 Amino acid descriptors

This encoding approach brings the physicochemical knowledge down to the amino acid level by representing a peptide as a sequence of amino acids, where each amino acid is encoded as predefined physicochemical properties. By default, this approach returns an L×18 dimensional list, where L is the number of amino acids in the peptide and a sub-selection of properties is possible.

### 2.3 BLOSUM62 encoding

BLOSUM62 is a matrix that contains amino acid similarities based on the reserved (sub)sequences on the phylogenetic trees (Eddy 2004). Similar to descriptors, BLOSUM62 also introduces domain knowledge to the model but encodes evolutionary information rather than physicochemical properties. BLOSUM62 encoding produces amino acid vectors such that each element in the vector encodes BLOSUM62 similarity score of one amino acid to another particular amino acid. In other words, BLOSUM62 vectors represent amino acids in terms of their similarity to other amino acids. peptidy integrates post-translational modifications to BLOSUM62 representation by adding a new binary dimension (optional). This function returns L×21 matrices by default, where 20 dimensions encode the standard amino acids and the added dimension encodes post-translations.

### 2.4 One-hot encoding

One-hot-encoding represents the peptide sequence in an n-dimensional vocabulary such that each dimension encodes the presence of a particular amino acid in a particular position (denoted with a 1 if present).

peptidy assigns a pre-defined position to each amino acid and post-translational modification in the vocabulary and represents the peptide sequences accordingly. This function returns a L×28 dimensional matrix by default, where each column encodes the existence of an amino acid or a post-translation, for a total of 28 elements in the dictionary.

### 2.5 Label encoding

Label encoding assigns a unique index (‘label’) to each sequence element and represents sequences as a (random) list of integers. When combined with deep learning, this encoding allows to learn optimal representations during training, starting from randomly initialized labels. This differs from one-hot encoding, where the vectors are fixed and pre-defined. Our implementation of label encoding supports the post-translational amino acid modifications.

## 3 Speed and memory usage

To test the usability of peptidy on a consumer computer (6 Intel i7-10700K CPU with 3.80 GHz clock, and 64 GB RAM), we analyzed its speed and memory usage. We generated 1 000 000 random amino acid sequences of length equal to 20 amino acids. On these sequences, we computed three descriptors (isoelectric point, molecular weight, and instability index) using peptidy, modlamp (Müller *et al.* 2017), and peptidespy (Larralde and Solomon, 2025). We calculated the average compute time and maximum memory used by each package, across 10 repetitions.

Computing these descriptors took around 10 min with peptidy, while with peptidespy and modlAMP it took 23 and 99 min on average, respectively (Table 2). These results highlight another advantage of peptidy, which is twice as fast as peptidespy and 10 times faster than modlAMP. The maximum memory usage is around 70 MB for all libraries, indicating that the speed of peptidy comes with no added memory cost.

**Table 2.** Speed and memory usage of different peptide processing libraries.^c^

Library	Speed (min)	Memory (MB)
peptidy	10.49±0.18	69.47±0.00
peptidespy ^a^	23.29±0.22	69.46±0.00
modlAMP ^b^	99.12±0.64	69.52±0.00

a Larralde and Solomon (2025).

b Müller *et al.* (2017).

c Three descriptors were computed on 1 M amino-acid sequences. Average speed and maximum memory usage across 10 repetitions were computed via time and tracemalloc Python modules, respectively. Single thread was used on a desktop with 16 Intel i7-10700K CPU (3.80 GHz) and 64 GB RAM.

## 4 Benchmarking peptide representations

We benchmarked peptidy representations using data available in the literature to predict (i) antimicrobial activity (Veltri *et al.* 2018) and (ii) toxicity (Wei *et al.* 2021) using machine learning. The amino acid sequences were divided into training (n=2000), validation (n=500), and test (n=240) splits for five repetitions (Monte-Carlo validation), while making sure that test sequences has at least 20% edit distance to training and validation sequences. Convolutional neural networks (LeCun *et al.* 1998) were trained on sequence representations, and XGBoost (Chen and Guestrin 2016) was selected to learn from the peptide descriptor representation. These machine learning models were chosen for their ideal trade-off between simplicity and performance (Özçelik and Grisoni 2025). One hundred hyperparameter combinations were screened per encoding strategy on each task, and the hyperparameter combinations yielding the best average validation loss were picked to predict the properties of the test set peptides. For the selected model, we computed the accuracy, precision, and F1 scores on the test sets (Table 3).

**Table 3.** Testing peptide encoding strategies available in peptidy.^a^

Property	Labels	No. peptides	Encoding	Accuracy (%)	Precision (%)	F1 (%)
Antimicrobial activity (Veltri *et al.* 2018)	Active/inactive	2740	Peptide descriptors	**90.33** ± **2.33**	88.16 ± 2.80	**87.93** ± **2.99**
			AA descriptors	86.42 ± 1.01	85.38 ± 2.00	82.46 ± 2.01
			BLOSUM62	89.42 ± 2.18	90.05 ± 5.17	86.22 ± 3.17
			One-hot encoding	88.83 ± 0.96	88.62 ± 1.55	85.58 ± 1.74
			Label encoding	90.25 ± 1.17	**91.42** ± **3.35**	87.25 ± 2.05
Toxicity (Wei *et al.* 2021)	Toxic/non-toxic	2740	Peptide descriptors	87.58 ± 1.70	88.90 ± 1.60	87.32 ± 1.57
			AA descriptors	79.75 ± 1.48	80.39 ± 2.01	79.38 ± 2.06
			BLOSUM62	**89.58** ± **2.04**	**91.82** ± **3.58**	**89.27** ± **1.97**
			One-hot encoding	89.00 ± 1.78	90.96 ± 3.75	88.75 ± 1.37
			Label encoding	88.50 ± 1.91	89.57 ± 2.84	88.32 ± 1.68

a Results are reported as the mean and standard deviation obtained across five training-test splits. The best and second-best scores for each metric and dataset are marked with boldface and underlining, respectively.

The obtained results—albeit computed on non-redundant training-test partitions determined in this study—are consistent with literature, which reports an accuracy of 91% for antimicrobial activity prediction (Veltri *et al.* 2018)), and accuracy values ranging from 87% to 95% for toxicity prediction (Wei *et al.* 2021). This supports the quality of the information captured by peptidy and its potential to be easily incorporated into machine learning pipelines.

Different types of encoding show different results depending on the metric and the task considered. For example, when predicting antimicrobial activity, global peptide descriptors produce the highest accuracy and F1 scores, while label encoding achieves the highest precision values. BLOSUM62, however, obtains the highest mean test set scores on the toxicity prediction task, followed by one-hot encoding. It is reasonable to assume that, for any new task, different representations might yield varying relative performances than what is shown in Table 3.

These results emphasize the value of experimenting with alternative representations when training peptide machine learning models. With its simplicity and flexibility, peptidy can facilitate such efforts by (i) ensuring consistency in evaluation, (ii) easily integrating into machine learning pipelines, and (iii) simplifying comparative analyses—ultimately accelerating the development and deployment of predictive tools for peptide sciences.

## 5 Discussion

peptidy aims to bridge the gap between peptide sequences and machine learning libraries by offering accessible encoding solutions out-of-the-box. peptidy not only makes popular encoding approaches as accessible as possible, it also extends the capabilities of available tools by supporting post-translational modifications that are critical to peptide properties. These elements, combined with its speed and the breadth of implemented representations, make peptidy particularly suited to lower the entry barriers into machine learning for peptides across many disciplines and areas of expertise.

peptidy is accompanied by extensive documentation and tutorials to facilitate accessibility. It is also open-sourced to allow feedback from researchers and extend its capabilities. We expect peptidy to be a useful tool for new machine learning researchers in the field.

## Data Availability

The peptidy package can be easily installed via pip install peptidy. The installation instructions, Python code, and benchmarking datasets to reproduce this study are available on GitHub at the following URL: https://github.com/molML/peptidy. The dataset and code to reproduce the analysis presented in Table 3 are available on Zenodo: https://zenodo.org/records/14945955.

## References

[vbaf058-B1] Chen T , Guestrin C. Xgboost: a scalable tree boosting system. In: *Proceedings of the 22nd ACM SIGKDD International Conference on Knowledge Discovery and Data Mining* ACM, San Francisco, California, USA, 2016, 785–94.

[vbaf058-B2] De Leon Rodriguez LM , Hemar Y. Prospecting the applications and discovery of peptide hydrogels in food. Trends Food Sci Technol 2020;104:37–48.

[vbaf058-B3] Eddy SR. Where did the BLOSUM62 alignment score matrix come from? Nat Biotechnol 2004;22:1035–6. 10.1038/nbt0804-1035 15286655

[vbaf058-B4] ElAbd H , Bromberg Y, Hoarfrost A et al Amino acid encoding for deep learning applications. BMC Bioinformatics 2020;21:235. 32517697 10.1186/s12859-020-03546-x PMC7285590

[vbaf058-B5] Erjavac I , Kalafatovic D, Mauša G. Coupled encoding methods for antimicrobial peptide prediction: how sensitive is a highly accurate model? Artif Intell Life Sci 2022;2:100034.

[vbaf058-B6] Fjell CD , Jenssen H, Hilpert K et al Identification of novel antibacterial peptides by chemoinformatics and machine learning. J Med Chem 2009;52:2006–15. 19296598 10.1021/jm8015365

[vbaf058-B7] Grisoni F , Neuhaus CS, Gabernet G et al Designing anticancer peptides by constructive machine learning. ChemMedChem 2018;13:1300–2. 29679519 10.1002/cmdc.201800204

[vbaf058-B8] Grisoni F , Neuhaus CS, Hishinuma M et al De novo design of anticancer peptides by ensemble artificial neural networks. J Mol Model 2019;25:112. 30953170 10.1007/s00894-019-4007-6

[vbaf058-B9] Hartmann R , Meisel H. Food-derived peptides with biological activity: from research to food applications. Curr Opin Biotechnol 2007;18:163–9. 17292602 10.1016/j.copbio.2007.01.013

[vbaf058-B10] Jenssen H. Descriptors for antimicrobial peptides. Expert Opin Drug Discov 2011;6:171–84. 22647135 10.1517/17460441.2011.545817

[vbaf058-B11] Larralde M , Solomon B. *GitHub—Althonos/peptides.py: Physicochemical Properties, Indices and Descriptors for Amino-acid Sequences—github.com*, 2025. https://github.com/althonos/peptides.py (28 February 2025, date last accessed).

[vbaf058-B12] LeCun Y , Bottou L, Bengio Y et al Gradient-based learning applied to document recognition. Proc IEEE 1998;86:2278–324.

[vbaf058-B13] Lee EY , Fulan BM, Wong GCL et al Mapping membrane activity in undiscovered peptide sequence space using machine learning. Proc Natl Acad Sci USA 2016;113:13588–93. 27849600 10.1073/pnas.1609893113 PMC5137689

[vbaf058-B14] Lee EY , Wong GCL, Ferguson AL. Machine learning-enabled discovery and design of membrane-active peptides. Bioorg Med Chem 2018;26:2708–18. 28728899 10.1016/j.bmc.2017.07.012 PMC5758442

[vbaf058-B15] Mangoni ML. Host-defense peptides: from biology to therapeutic strategies. Cell Mol Life Sci 2011;68:2157–9. 21584810 10.1007/s00018-011-0709-3 PMC11114633

[vbaf058-B16] Müller AT , Gabernet G, Hiss JA et al modlamp: Python for antimicrobial peptides. Bioinformatics 2017;33:2753–5. 28472272 10.1093/bioinformatics/btx285

[vbaf058-B17] Osorio D , Rondón-Villarreal P, Torres R. Peptides: a package for data mining of antimicrobial peptides. Small 2015;12:44–444.

[vbaf058-B18] Özçelik R , Grisoni F. A hitchhiker’s guide to deep chemical language processing for bioactivity prediction. Digit Discov 2025;4:316–25. 39726698 10.1039/d4dd00311j PMC11667676

[vbaf058-B19] Sharma K , Sharma KK, Sharma A et al Peptide-based drug discovery: current status and recent advances. Drug Discov Today 2023;28:103464. 36481586 10.1016/j.drudis.2022.103464

[vbaf058-B20] Veltri D , Kamath U, Shehu A. Deep learning improves antimicrobial peptide recognition. Bioinformatics 2018;34:2740–7. 29590297 10.1093/bioinformatics/bty179 PMC6084614

[vbaf058-B21] Wei L , Ye X, Xue Y et al ATSE: a peptide toxicity predictor by exploiting structural and evolutionary information based on graph neural network and attention mechanism. Brief Bioinform 2021;22:bbab041. 33822870 10.1093/bib/bbab041

[vbaf058-B22] Yue Z-X , Yan T-C, Xu H-Q et al A systematic review on the state-of-the-art strategies for protein representation. Comput Biol Med 2023;152:106440. 36543002 10.1016/j.compbiomed.2022.106440

